# Impact of spectral body imaging in patients suspected for occult cancer: a prospective study of 503 patients

**DOI:** 10.1007/s00330-020-06878-7

**Published:** 2020-05-04

**Authors:** Michael Brun Andersen, Dyveke Ebbesen, Jesper Thygesen, Matthijs Kruis, Finn Rasmussen

**Affiliations:** 1 Department of Radiology, Copenhagen University Hospital Herlev and Gentofte, Gentofte Hospitalsvej 1, 2900 Hellerup, Denmark; 2 Department of Radiology, Zealand University Hospital Roskilde, Sygehusvej 10, Roskilde, 4000 Denmark; 3 Department of Radiology, Aarhus University Hospital, Palle Juul-Jensens Blvd. 161, Aarhus, 8200 Denmark; 4 Department of Clinical Engineering, Central Denmark Region, Nørrebrogade 44, Building 2A, Aarhus, 8000 Denmark; 5 Philips Medical Systems, Clinical Science, CT, Veenpluis 4-6, Best, 5684 The Netherlands

**Keywords:** Helical CT, Clinical oncology, Thorax, Abdomen

## Abstract

**Objectives:**

To investigate the diagnostic impact and performance of spectral dual-layer detector CT in the detection and characterization of cancer compared to conventional CE-CT.

**Methods:**

In a national workup program for occult cancer, 503 patients (286 females and 217 males) were prospectively enrolled for a contrast-enhanced spectral CT scan. The readings were performed with and without spectral data available. A minimum of 3 months between interpretations was implemented to minimize recall bias. The sequence of reads for the individual patient was randomized. Readers were blinded for patient identifiers and clinical outcome. Two radiologists with 9 and 33 years of experience performed the readings in consensus. If disagreement, a third radiologist with 11 years of experience determined the outcome of the reading

**Results:**

Significantly more cancer findings were identified on the spectral reading. In 73 cases of proven cancer, we found a sensitivity of 89% vs 77% and a specificity of 77% vs 83% on spectral CT compared to conventional CT. A slight increase in reading time in spectral images of 82 s was found (382 vs 300, *p* < 0.001). For all cystic lesions, the perceived diagnostic certainty increased from 30% being completely certain to 96% most pronounced in the kidney, liver, thyroid, and ovaries. And adding the spectral information to the reading gave a decrease in follow-up examination for diagnostic certainty (0.25 vs 0.81 per reading, *p* < 0.001).

**Conclusion:**

The use of contrast-enhanced spectral CT increases the confidence of the radiologists in correct characterization of various lesions and minimizes the need for supplementary examinations.

**Key Points:**

• *Spectral CT is associated with a higher sensitivity, but a slightly lower specificity compared to conventional CT.*

• *Spectral CT increases the confidence of the radiologists.*

• *The need for supplementary examinations is decreased, with only a slight increase in reading times.*

**Electronic supplementary material:**

The online version of this article (10.1007/s00330-020-06878-7) contains supplementary material, which is available to authorized users.

## Introduction

Computed tomography (CT) is the leading modality for the detection and characterization of neoplasms [1]. Iodine is commonly used as a contrast agent, because it provides great CT contrast and can relatively safely be injected into the bloodstream [2]. The fact that perfusion is considered a hallmark of cancer biology [3] makes iodine contrast-enhanced CT a very potent modality for cancer diagnoses.

Developments in dual-energy or spectral CT have been a major leap for CT. Most techniques create a dual-energy dataset by scanning the same area twice with different kilovolt peak/filtration settings. This can be achieved either by performing two completely separate acquisitions, by rapidly alternating the kilovolt peak during the rotation, or by using two x-ray sources and detectors in one CT scanner. The latest commercial spectral solution uses a dual-layer detector to separate the x-ray spectrum, without the need for changing the tube kilovolt peak or any other scanning parameters. The top row detects low-energy photons while the bottom layer detects high-energy photons [4].

Spectral CT improves quantification of materials and characterization of tissue in comparison to conventional single-energy CT. Where conventional CT provides Hounsfield units (HUs) that describe the scanned tissues relative x-ray attenuation properties for a particular tube output spectrum, spectral CT provides quantitative measures like material decomposition, material concentrations, and suppression of signals associated with specific materials. There is a wide variety of materials to which this can be applied like iodine, water, calcium, uric acid, fat, and iron [5, 6].

Improvement of iodine quantification and visualization with spectral CT has shown improved detection and characterization of a wide variety of neoplasms [7]. Examples of these are improvements in lung nodule characterization [8], hypervascular liver lesion detection [9, 10], differentiation between malignant and benign thyroid nodules [11, 12], characterization of lesions as simple cysts in the kidneys [13–15], adrenal adenoma and metastasis differentiation [16, 17], hypodense pancreatic lesion delineation [18], detection of iso-attenuating pancreatic lesions [19, 20], and differentiation of prostate carcinoma from benign hyperplasia [21].

In Denmark (DK), the National Board of Health (NBH) has defined health packages for diagnosis and treatment of specific diseases and patient groups, to streamline diagnoses and treatment. Furthermore, it ensures that all patients are treated according to national guidelines.

Among these packages, 28 specific cancer treatment pathways are implemented. With specific cancer symptoms, patients will be directed to specific pathways. However, when a patient presents to the general practitioner with a set of serious, but more vague, cancer-indicating symptoms (e.g., fatigue, pain, fever, unintended weight loss, abnormal blood chemistry), the patient can be referred to a package for suspected serious illness that could be cancer (occult cancer package). The incidence of cancer for patients enrolled in this package lies around 16% and based on recommendation from the National Board of Health, whole body iodine contrast-enhanced CT is the imaging modality of choice in such patients [22].

The purpose of this study is to evaluate the impact of contrast-enhanced spectral CT (CE-SCT) compared to contrast-enhanced CT (CE-CT) in a prospectively gathered cohort of patients that enter the occult cancer pathway. The hypothesis is that CE-SCT will detect more lesions than CE-CT, that CE-SCT is better in the characterization of a lesion than CE-CT, and that the confidence level of the reporting radiologist increases using CE-SCT compared to CE-CT. Furthermore, workflow and need for supplementary diagnostics will also be presented.

## Materials and methods

### Patients

Five hundred thirty-six patients, referred by their general practitioner (GP) to the occult cancer pathway, were prospectively included in the study between May 2017 and November 2018. The ethical committee waived ethical issues and the study was approved by the Danish data authorities. Five hundred three patients gave written consent to participate in the project, and allowed access to image and clinical data.

**Fig. 1:**
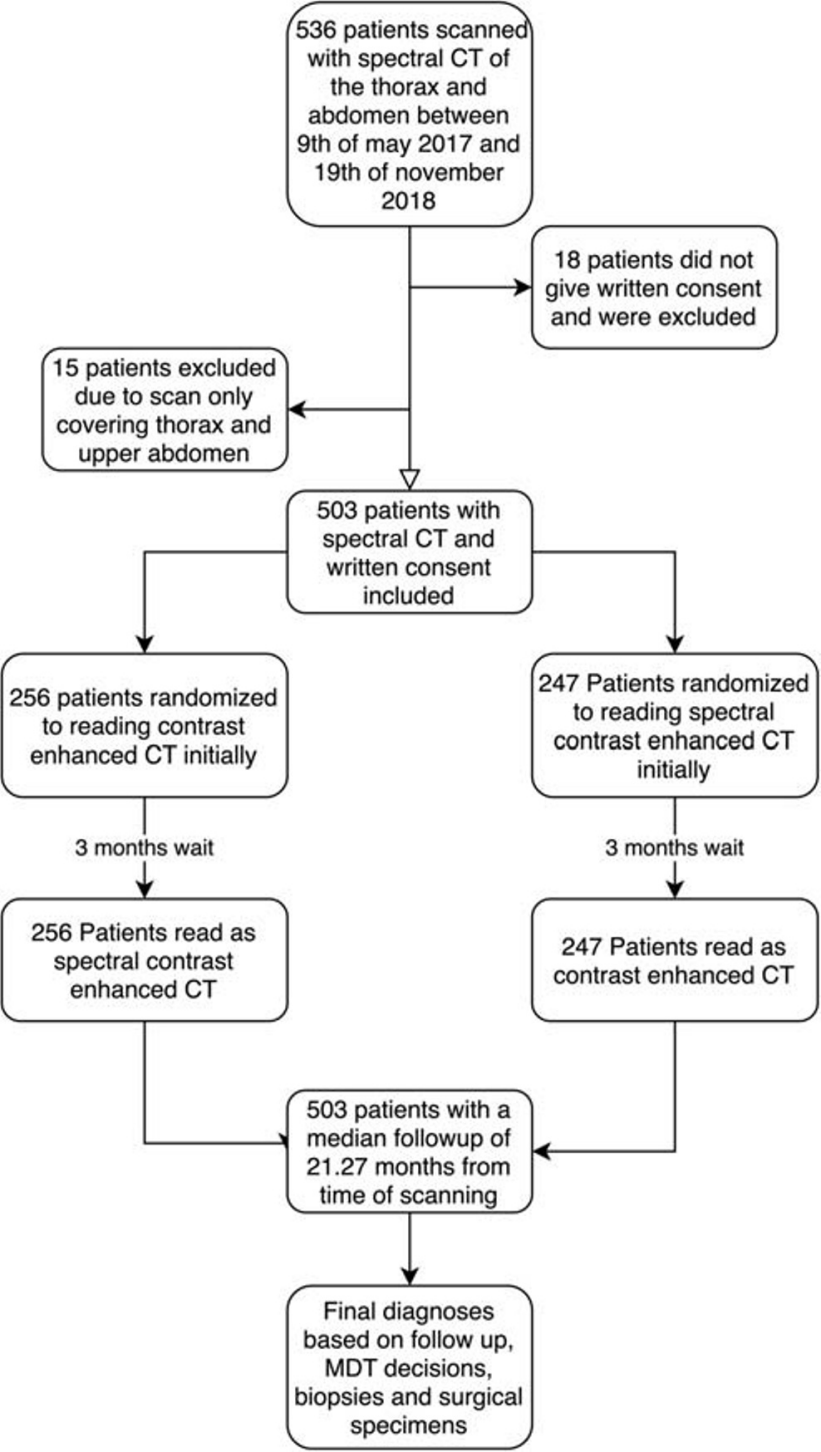
A flowchart of inclusion, exclusion, and the overall study design

Patients referred from the GP to the fast track package for suspected serious illness that could be cancer were eligible for inclusion. Exclusion criteria were missing written consent, scan protocol differing from the national guidelines, and allergies to iodine contrast media. Figure 1 provides an overview of the study design.

Patient in the study population presented themselves to the GP with numerous symptoms and risk factors (see Table 1). Unfortunately, the referral information is often incomplete.

**Table 1:** Symptoms presented by the patients to the general practitioner and used in the referral for the workup program. The list also reports the number of smokers and patients with an earlier cancer diagnosis

Symptoms/risk factors	*n*
Weight loss	240
Pain	185
Fatigue	181
Diarrhea	87
Dyspepsia	73
Anemia	72
Coughing	61
Nightly sweats	59
Increased liver parameters	53
Dyspnea	49
Palpable tumor	35
Increased abdominal width	29
Back pain	14
Smoking	61
Earlier diagnosed with cancer	60

An overview of the basic demographics is included in Table 2.

**Table 2:** Patient demographics: gender, age, weight, height, radiation dosage, and patient categories

	*n*	Mean	Std. dev.	95% confidence interval	Min	Max
Males	216					
Females	287					
Age (year)	503	64.48	13.20	53.29–65.67	23	90
Height (cm)	467	171.61	8.80	170.81–172.41	147	196
Weight (kg)	469	73.53	15.64	72.11–74.95	33	150
Dose length product (mGy × cm)	503	2104.12	441.91	2064.24–2144.00	1375.9	4778
Proven cancer	73					
Likely cancer based on imaging	15					
Unlikely cancer	31					
Other proven serious disease	22					
Proven benign	152					
No finding described in follow-up	210					

### Spectral contrast-enhanced CT and contrast-enhanced CT

SCE-CT of the chest, abdomen, and pelvis was acquired on a 64-row dual-layer detector CT scanner (Philips IQon; Philips Healthcare). CT acquisition parameters were 64 × 0.625-mm collimation, kilovolt peak 120–140, milliamperes per second/slice 150–250, rotation time 0.75 s, reconstruction thickness 2 mm, increment 1 mm, pitch 1.078, FOV 35 cm, and matrix 512 × 512. Iodixanol 270 mg/ml (Visipaque® 270; GE Healthcare), or iohexol 300 mg/ml (Omnipaque® 300; GE Healthcare), was injected intravenously in weight-adjusted doses of 2 ml/kg body weight to compensate for differences in distribution volume, with an injection rate of 4 ml/s. A bolus tracking technique was used with a ROI in the descending aorta on the level of carina to compensate for differences in cardiac output. A threshold of 150 HU was used and CT was performed after a delay of 15 s for the chest and upper abdomen (late arterial phase), and 65 s for the abdomen (portal venous phase). The mean dose length product (DLP) of CT scans performed on the population was 2104 mGy·cm (CI95% 2064 to 2144). By spectral separation of the CT signal in the two detector layers, a spectral CT dataset was reconstructed. By weighted addition of the signal of the two layers together before reconstruction, a conventional CT dataset was reconstructed that possesses all features of a normal single energy CT in terms of dose [23] and image quality [24].

### Reading of examinations

Scan data were transferred to a dedicated spectral workstation (Intellispace 9; Philips Healthcare) and divided into primary readings and secondary reading folders. The reading folders contained either the CE-CT or a superset of the conventional results and the CE-SCT. The provided spectral data include the following: virtual monoenergetic images (ranging from 40 to 200 keV), effective atomic number (*Z*_eff_, reporting the atomic number of the tissue), iodine-no-water (pure spectral decomposition of iodine and water. Calcium remains visible and is mimicked by iodine), iodine density (similar to iodine no water, but calcium is masked out), contrast enhancing structures (masking of iodinated tissues), uric acid (masking of uric acid containing tissue), and virtual non-contrast (VNC, a 70 keV map without the signal of iodine contrast).

Overlay images on the conventional series or the VNC was available for all datasets.

The scans were hereafter read in consensus by two experienced radiologists, with respectively 9 and 33 years of experience. In case of disagreement, a third radiologist would determine the outcome of the proposed findings.

All organ systems covered in the scans were reviewed. Initial reading was performed with virtual monoenergetic 40 keV to identify lesions that required further attention. In suspicion of disease, other spectral results were investigated. For instance, VNC was used in the case of a suspicion of calcification to discriminate calcium from iodine. In contrary, for low- or hyperattenuated lesions, iodine density, iodine no water, contrast enhancing structures, and *Z*_eff_ were used to prove the presence of iodine.

To prevent recall bias, the primary and secondary reading were performed with an interval of at least 3 months and in random order. The radiologists were blinded from patient identifiers and earlier imaging, but had access to symptoms and a concise medical background of the patient mentioned in the referral.

All findings were entered into a RedCAP database [25]. Up to 7 findings per patients were classified according to their severity and were scored for malignancy of the finding (1 = “certainly malignant,” 2 = “probably malignant,” 3 = “probably benign,” and 4 = “certainly benign”) and certainty of the finding (1 = “certain,” 2 = “almost certain,” 3 = “somewhat uncertain,” and 4 = “very uncertain”).

During the course of the study, we decided to record additional information. For a subset of the readings (308 spectral and 304 conventional), we recorded the reading time. For another subset (418 spectral and 414 conventional), we recorded the need for supplementary examinations. For the last 221 patients (221 spectral and 221 conventional readings), we also recorded whether the indicated supplementary examinations were part of routine or needed to improve the certainty of the finding.

Routine follow-up procedures included a wide range of examinations, including CT thorax in case of pulmonary nodules according to Fleischner Society criteria, multiphase CT or ultrasound in case of hyperdense lesions in the kidney, PET/CT in the case of suspected lung cancer, transvaginal ultrasound for ovarian lesions, dedicated adrenal CT for adrenal lesions, ultrasound and possible biopsy from thyroid lesions, etc. In the case of uncertainty, the follow-up procedures include contrast-enhanced ultrasound in liver or kidney lesions, biopsy or follow-up on enlarged/suspicious lymph nodes, endoscopies when intraluminal lesions, or mural thickening was suspected etc.

### Review of findings

Individual findings were matched between the two readings, according to disease type and associated organ.

After a median follow-up of 21.3 months, the clinical status was recorded for every patient. When the clinical status could not validate a recorded serious first finding (cancer or another disease that would need immediate medical treatment), a more thorough search in the medical records was conducted for validation. When a serious finding was not considered in the medical history of the patient, the case was reported to the Ethical Committee for additional follow-up.

Every patient would now have an outcome value (1 = “cancer, with recorded proof,” 2 = “likely cancer, without recorded proof,” 3 = “unlikely cancer,” 4 = “proven benign,” − 1 = “finding not investigated,” O = “other proven serious disease”).

Most of the benign findings were not reported in the patient dossier and would get a score of “− 1.” There were also potential malignant findings without follow-up that received a score of “2” or “3.” Very unlikely cancers were often not investigated and patients often did not want follow-up diagnostics or were not able to cooperate/receive the suggested procedure. Other serious diseases (“O”) were conditions that required treatment within a short period of time.

Cancer, with recorded proof, was obtained by the following methods: biopsy in 48 cases, surgery in 9 cases, multidisciplinary team decision in 11 cases, follow-up in 2 cases, and in 2 cases the patient had a diagnosed cancer not reported in the initial referral.

### Statistical analysis

We hypothesized that CE-SCT would detect more findings than CE-CT. We used McNemar’s test to identify types of findings that differed in frequency between the two modalities.

To test the hypothesis that spectral findings provide a higher confidence to the radiologist in the diagnoses compared to conventional findings, we used a two-sided two proportion *z*-test to find the types of findings for which the proportion of certain and uncertain findings differed between CE-SCT and CE-CT.

We compared sensitivity and specificity of malign findings between CE-SCT and CE-CT and tested for significance by means of McNemar’s test.

We performed descriptive statistics and box-and-whisker plots for the reading time per patient of both CE-SCT and CE-CT. Differences in reading time between CE-SCT and CE-CT based on the certainty of the first finding were also compared using a box-and-whisker plot.

Furthermore, we compared the frequency of supplemental procedures between CE-SCT and CE-CT by means of a two-sided *t* test.

## Results

There were slightly more findings reported on the spectral than the conventional readings (6.8 vs. 6.4 findings per patient, *p* < 0.001). An overview of specific diseases with significantly different frequencies can be found in Table 3.

**Table 3:** Organs/findings with a significant difference in incidence between the conventional contrast-enhanced CT and the spectral contrast-enhanced CT

Organ	Description of finding	Found on both modalities	Number of patients with no finding	Found on SCE-CT	Found on CCE-CT	*p* value McNemar’s test
Pancreas						
	Cancer/tumor	8	489	6	0	0.04
Prostate						
	Process/localized enhancement	3	443	57	0	< 0.001
	Hypertrophy	44	397	12	50	< 0.001
Adrenal						
	Adenoma	1	473	28	1	< 0.001
	Incidentaloma	10	449	10	34	< 0.001
Thyroid						
	All findings (cysts, nodules, etc.)	86	353	44	20	< 0.001
	Thyroid nodule	13	440	39	11	< 0.001
Biliary						
	All findings (stones, polyps, etc.)	63	403	26	11	0.02
	Polyp	0	493	10	0	< 0.001
Heart						
	Coronary calcification	39	359	88	17	< 0.001

Table 4 lists the frequencies of various types of findings with significant differences in certainty.

**Table 4:** The frequency of various findings with significantly differences in certainty, as indicated by the radiologists in the readings

Finding type	Location of finding	Completely certain SCE-CT	Uncertain SCE-CT	Completely certain CCE-CT	Uncertain CCE-CT	*p* value *Z* test
All findings	Overall	2415	158	1920	522	< 0.001
	Kidney	271	12	117	153	< 0.001
	Liver	269	30	126	164	< 0.001
	Thyroid	134	5	54	46	< 0.001
	Adrenal	56	0	49	6	0.011
Cysts	Overall	474	20	142	331	< 0.001
	Kidney	203	7	60	141	< 0.001
	Liver	149	6	26	139	< 0.001
	Thyroid	65	1	23	32	< 0.001
	Ovaries	28	1	19	5	0.047
Process (tumor, nodule, neoplasm)	Overall	136	16	56	25	< 0.001
	Liver	20	5	8	8	0.044
	Thyroid	37	2	12	11	< 0.001
	Prostate	52	3	0	1	< 0.001
Calcifications	Coronary vessels	109	0	31	10	< 0.001

In the patient population, 73 proven cancers were observed together with 22 other serious diseases (Table 2). Imaging characteristics for the proven cancers are shown in Table 5. The sensitivity for finding these 73 cancer cases was higher in the spectral than in the conventional readings (89% vs. 77%, *p* < 0.005).

**Table 5:** Imaging characteristics for the 73 confirmed malignant lesions

	*N*	Number of lesions unmeasurable on CT	Average size (mm)	Average conventional attenuation (HU)	Average attenuation 40 keV (HU)	Average attenuation 200 keV (HU)	Average iodine concentration (mg/ml)	Average effective atom number (*Z*_eff_)
Colon	5	3	49 (std. dev. 8.9)	73.7 (95% − 247.2 to 394.4)	262 (95% − 525.8 to 1049.8)	39.15 (95% − 21.2 to 99.5)	1.5 (95% − 9.9 to 12.9)	8.05 (95% 2.3 to 13.8)
Esophagus	1	0	26	72.1	158.4	46.5	1.4	8.1
Intestines	3	0	59 (std. dev. 17.1)	69.3 (95% 3.15 to 135.38)	153.7 (95% − 20.8 to 328.2)	35.5 (95% 6.2 to 64.7)	1.5 (95% − 2.2 to 3.2)	8.06 (95% 7.0 to 9.1)
Kidney	4	0	20 (std. dev. 13.5)	122.4 (95% 56.2 to 188.6)	307.7 (95% 30.0 to 585.3)	54.7 (95% 15.9 to 93.5)	3.1 (95% − 0.5 to 6.7)	8.85 (95% 7.2 to 10.4)
Liver	4	0	101 (std. dev. 34.4)	68.5 (95% 37.9 to 99.1)	153.5 (95% 54.7 to 252.2)	44.1 (95% 32.0 to 56.1)	1.4 (95% 0.3 to 2.4)	8.03 (95% 7.4 to 8.6)
Lungs	16	0	34 (std. dev. 23.9)	− 29.2 (95% − 147.7 to 89.2)	65.4 (95% − 54.3 to 185.1)	− 61.9 (95% − 183.7 to 59.9)	1.6 (95% 1.2 to 2.0)	8.33 (95% 7.9 to 8.7)
Lymph nodes	9	0	53 (std. dev. 39.6)	75.3 (95% 63.5 to 87.1)	171.2 (95% 135.8 to 206.6)	40.3 (95% 34.1 to 46.5)	1.6 (95% 1.2 to 2.0)	8.16 (95% 7.9 to 8.4)
Mamma	2	0	16 (std. dev. 5.1)	44.9 (95% − 0.8 to 90.6)	49.5 (95% − 83.3 to 182.2)	44.5 (95% 12.7 to 76.3)	0.1 (95% − 1.2 to 1.4)	7.3 (95% 6.0 to 8.6)
Musculosceletal	1	0	12	69.9	130.1	49.4	0.6	7.8
Ovary	1	0	38	42	92.8	30.8	0.8	7.7
Pancreas	12	0	38 (std. dev. 23.4)	65.9 (95% 53.8 to 78.1)	156.2 (95% 121.2 to 191.3)	32.8 (95% 28.5 to 37.1)	1.5 (95% 1.2 to 1.9)	8.14 (95% 7.9 to 8.3)
Prostate	10	1	21	86.8 (95% 72.9 to 100.7)	214.7 (95% 167.5 to 261.9)	47.2 (95% 43.2 to 51.2)	2.1 (95% 1.5 to 2.6)	8.37 (95% 8.1 to 8.6)
Spleen	1	0	128	139.3	414.5	56.5	4.3	9.3
Carcinosis	2	0	20 (std. dev. 13.6)	65.6 (95% − 35.5 to 166.6)	146.9 (95% − 292.1 to 585.8)	33.7 (95% − 3.8 to 71.1)	1.4 (95% − 4.9 to 7.8)	8.05 (95% 4.9 to 11.2)
Uterus	1	0	171	54.4	102.7	37.4	0.8	7.8

The specificity of the cancer findings was slightly smaller in the spectral findings (77% vs. 83%, *p* < 0.005). The main reason for this difference was 18 possible prostate cancer findings without follow-up. We found a lower positive predictive value (40% vs 43%), but a higher negative predictive value (98% vs 96%) for CE-SCT.

The reading time differed significantly between the spectral readings (see Electronic Supplementary Material). The radiologists spent on average 382 s analyzing the spectral readings versus 300 s at the conventional readings. The difference in reading time between the spectral and the conventional reading was largest when the first finding of the spectral reading was uncertain. When the first finding on the spectral reading was certain, the difference was much smaller.

Although more findings were defined during the spectral reading, we found a significantly lower number of requested follow-up procedures per reading (for spectral 0.91 and for conventional 1.39 follow-ups per reading, *p* < 0.001). For the last 221 patients, we also distinguished between follow-ups for clinical routine and follow-ups needed for diagnosis certainty. We found a decrease in the need for follow-ups for diagnosis certainty (0.25 vs. 0.81 follow-ups per reading, *p* < 0.001). The numbers of clinical routine follow-ups did not differ significantly between the two readings (0.62 vs. 0.66, *p* = 0.43).

## Discussion

Godfrey Hounsfield proposed the concept of using multiple energies in computed tomography to perform material decomposition already in 1973 [26]. However, the first clinical CT scanner that could perform both dual-energy series in one rotation was developed in 2008 as a dual source system [27]. Although many studies have indicated a clinical advantage in a wide variety of applications, clinical adaptation has been lacking due to an associated increase in reading time, additional data, and reconstruction time compared to the CE-CT [28].

In the early iterations of dual-energy CT/spectral CT, increased radiation dose was a major concern [29]. With the current scanner technology, the dose has been decreased and the concern is outweighed by the benefits associated with spectral CT [30]. In this study, the mean dose measured by DLP was 2104 mGy·cm (CI95% 2064 to 2144, range from 1081 to 4778). A total of 28 patients had a BMI of < 18.5 (mean 24.9 CI95% 24.5 to 25.3, range from 13.2 to 55.1) and were considered underweight. In a standard setting with CE-CT, they would most likely have been scanned with 100 kVp; however, none of the present dual-energy/spectral CT scanners are capable of producing images of diagnostic quality and provide dual-energy information at doses comparable to single-energy 100-kVp images. It corresponds to roughly 5% of the population described in our study and in our opinion the benefits outweigh the minor increase in average patient dose.

In this study, a scanner with a dual-layer detector that is able to perform spectral acquisitions, without changing the acquisition protocol, was used. The reconstruction of the Spectral Base Images took typically less than 5 min, after which all spectral results were instantly available to the radiologist, without the need for additional reconstructions at the workstation.

Due to this integrated solution, the additional mean spectral reading time was limited to an increase of 82 s compared to CE-CT (see Electronic Supplementary Material). It is important to acknowledge that these reading times are associated to the specific system, workflow, and setup at our institution and that it was done in a research setting with no distractions. Reading times might differ with other dual-energy CT/spectral CT systems and institutional setups, and should be investigated thoroughly.

**Fig. 2:**
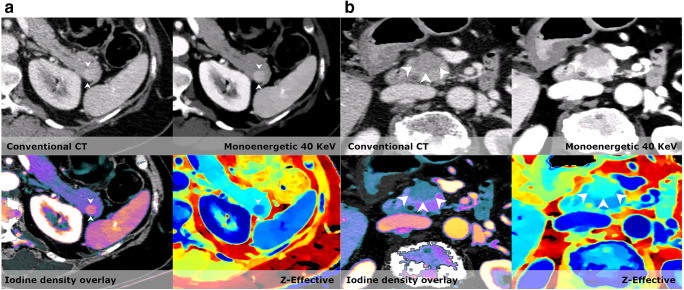
**a** In the pancreatic tail, a small lesion (arrowhead) is barely visible on the conventional CT. However, on all spectral series, the lesion becomes evident. The enhancement corresponds to the spleen and differential diagnoses were both an intrapancreatic accessory spleen and a small neuroendocrine tumor. Historic data of the patient however demonstrated that the lesion had not changed in 12 years, and therefore this lesion was characterized as an intrapancreatic accessory spleen. **b** A patient with a slight increase in volume in the head of the pancreas and stranding in the peripancreatic fat. In the pancreatic head, a slight hypodense lesion (arrowheads outline the lesion) is visible on the conventional image; however, it is easily missed. On all spectral images, the lesion stands out against the normal pancreatic parenchyma and the lesion was correctly diagnosed as a pancreatic adenocarcinoma

Numerous publications on the use of spectral CT for the detection and characterization of neoplasms have been published over the past decade. Most publications only include small and selected patient populations [28]. The aim of this study was to implement current knowledge into a daily clinical routine on a patient population with a high probability of cancer. The major outcome of this study was a higher observed sensitivity of CE-SCT compared to CE-CT when malignancy is considered (89% vs. 77%, *p* < 0.005), albeit with a slightly lower observed specificity (77% vs. 83%, *p* < 0.005). The main reason for this decrease in specificity was prostate findings, of which many were not further investigated. The increase in sensitivity and corresponding drop in specificity can somewhat be compared to other imaging modalities like positron emission tomography/CT (PET/CT), which also have a high sensitivity at the cost of an increased number of false positive findings.

In this study, we have 8 false negatives for CE-SCT comprised of 2 colon cancers, 2 pancreatic cancers, and 2 prostate cancers, even retrospectively 4 of the lesions are not found. The remaining 2 lesions were detected, but categorized as benign: a ground-glass lung nodule, followed up correctly, and a cancer mamma with no enhancement on iodine quantification.

The perceived certainty in diagnoses, especially for benign cystic lesions, increased from 30% being certain to 96%, and adding the spectral information to the reading will allow a decrease in total number of follow-up examinations from 1.39 to 0.91 per exam.

The differences in sensitivity, specificity, and general certainty were concentrated within a number of specific organs: pancreas, prostate, thyroid, kidney, and adrenal glands.

Virtual low monoenergetic reconstructions increase the ability to detect and stage pancreatic cancer. In recent studies, optimal contrast-to-noise ratio settings have been determined to be 40 keV and optimal image quality at 55–65 keV [18]. By using these settings as part of our routine assessment of the pancreas in CE-SCT series, we found significantly more suspicious lesions in the pancreas than on CE-CT (14 vs. 8). Because the readers were blinded to prior examinations, some lesions found on spectral were wrongly perceived as being potentially malignant. An example of this was a case showing a roughly 1.3-cm vague hyperdense lesion in the pancreatic tail that was missed on the conventional series (Fig. 2a). Based on the available imaging, it could represent both an intrapancreatic accessory spleen and a pancreatic neuroendocrine tumor. However, historic records of this patient described this as an intrapancreatic spleen and this lesion had been stable for over 5 years. In another patient, a definitive malignant lesion was suspected on the conventional series but became evident on CE-SCT series (Fig. 2b); the lesion later proved to be a small adenocarcinoma.

**Fig. 3:**
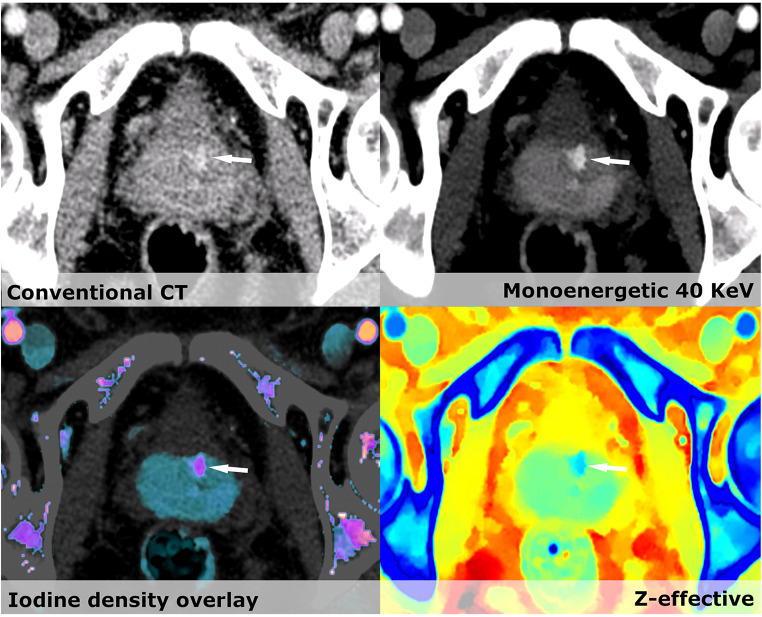
Small lesions, like this small lenticular lesion (arrow) < 1.5 cm in size, are often overlooked. The lesion is easy to see on both virtual low monoenergetic images, iodine density overlays, and Z-effective. It is anteriorly located on the edge of the transition zone. A biopsy confirmed that this lesion was a prostate cancer

For prostate imaging, MRI is currently the imaging modality of choice. However, studies on prostate CE-CT have suggested that areas of mass-like enhancement in the periphery of the prostate on venous phase may correlate with neoplasms and should warrant further follow-up [21]. In our patient cohort, the contrast enhancement in the periphery is potentiated by using virtual low monoenergetic images. We found significantly more focal lesions within the prostate using CE-SCT compared to CE-CT. The reason for finding significantly more hypertrophic prostates on CE-CT is that in case of focal enhancement, prostate hypertrophy was not reported (Fig. 3). Unfortunately, the diagnostic impact of these changes seen on CE-SCT in the prostate gland could not be validated in the current setup. Comparative studies between CE-SCT with state-of-the-art MRI of the prostate are needed [31, 32].

**Fig. 4:**
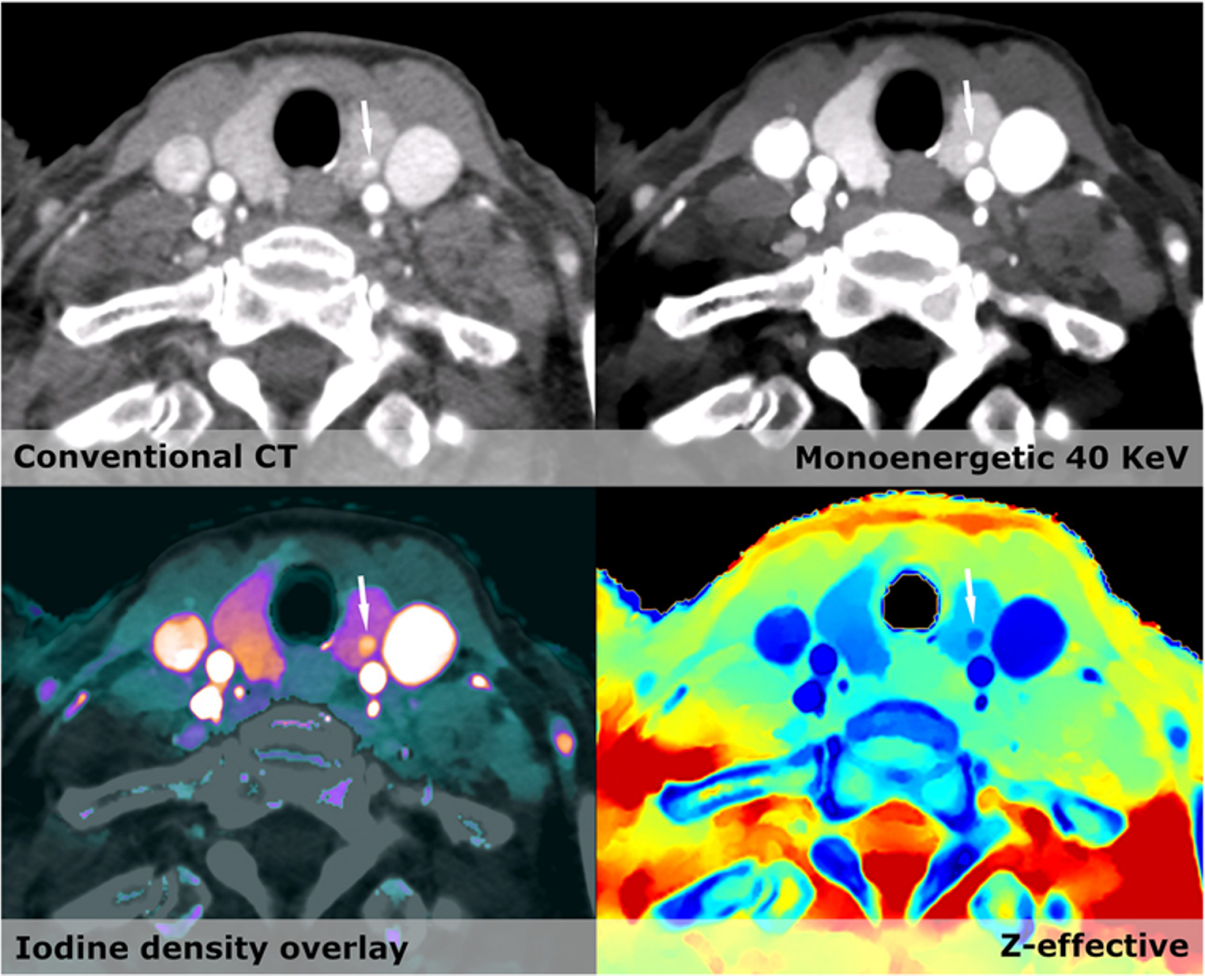
On all images, a small lesion (Arrow) in the left thyroid lobe is seen. However, using the spectral information, the lesion is most likely benign with high iodine density of > 6 mg/ml

Adrenal gland lesions most often represent benign adenomas, but the presence of iodine makes differentiation a challenge on CE-CT. From earlier studies [16, 17], we know that adrenal adenomas can be confidently identified on VNC by using a cutoff value of < 10 HU with a relatively high sensitivity of 73% and a specificity of 100%. This made it possible in the current cohort to evaluate enlarged adrenal glands and confidently characterize them in a majority of the cases, minimizing the need for added follow-up examinations. We found significantly more adrenal adenomas with CE-SCT, which related to an increase of adrenal incidentalomas found on CE-CT [16, 17].

Management of thyroid nodules on CE-CT has always been challenging. One study found an increase in iodine concentration and *Z*_eff_ was able to distinguish papillary carcinomas from benign multinodular goiter [11, 12]. We observe significantly more lesions by CE-SCT and can better characterize actual iodine enhancement within them using iodine density and *Z*_eff_ (Fig. 4). However, the evidence is scarce and will require a study design constructed to distinguish between benign and suspicious lesions.

**Fig. 5:**
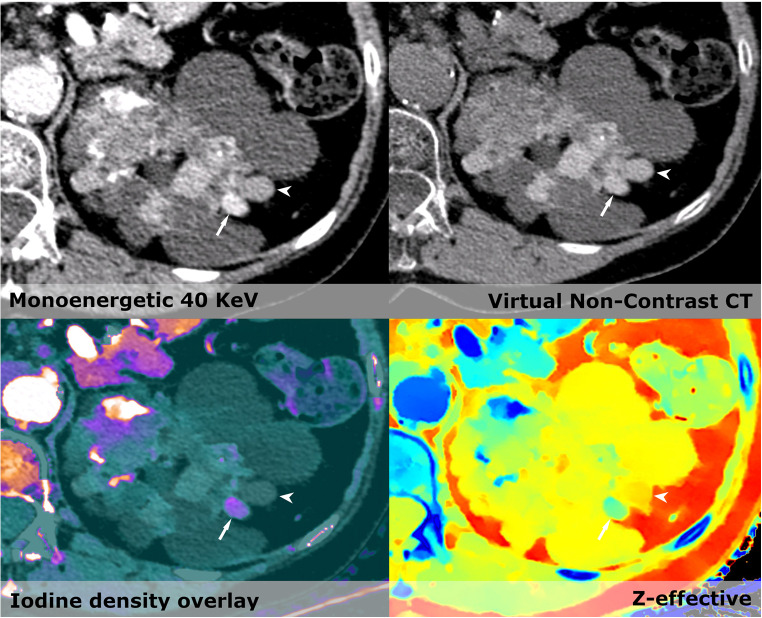
Conventional CT shows several hyperdense lesions within the kidney besides the simply cysts. VNC shows differences in several areas, most markedly within one of the two hyperdense lesions (Arrow) in the lateral part of the kidney. Iodine density overlay and Z-effective show a clear enhancement in the area mentioned. Next to this is a hyperdense cyst (arrowhead) with no enhancement and Z-effective values below 8. The diagnosis are most likely renal carcinoma within a polycystic kidney

Incidental renal masses are very common and difficult to assess without a non-contrast CT. Effective atomic number (*Z*_eff_) using a cutoff value of 8.36 can be used to distinguish between true enhancing and non-enhancing renal masses [10]. Even though we did not find significantly more enhancing masses in the kidneys on the CE-SCT compared to the CE-CT, the confidence level of our diagnoses of cysts was dramatically increased, therein also minimizing the need for supplementary examinations. In a case of polycystic kidney disease, CE-SCT made the reading markedly easier as it could readily distinguish between true enhancement, calcifications, hyperdense cysts, and simple cysts. CE-SCT showed a hyperdense cyst and a contrast enhancing tumor within the left kidney. CE-CT merely shows two hyperdense lesions (Fig. 5).

**Fig. 6:**
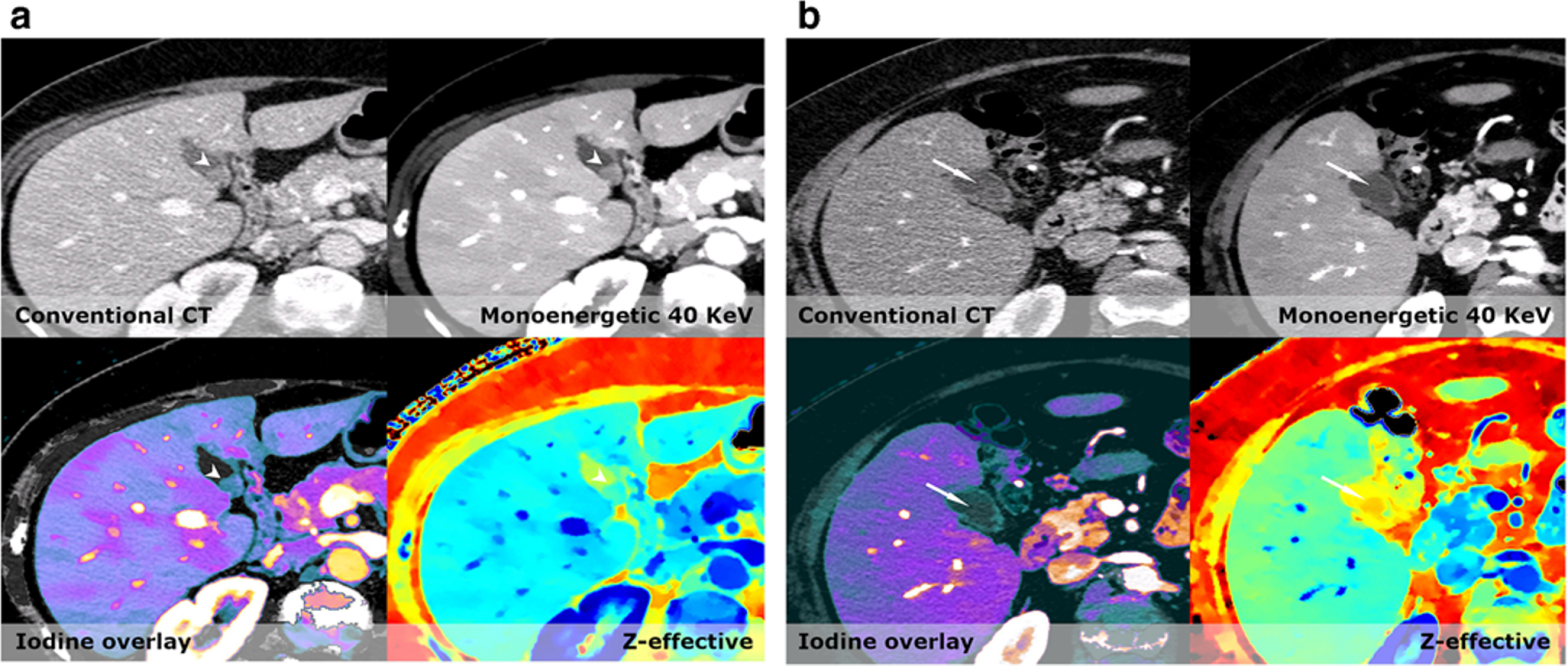
**a** Conventional CT shows a hyperdense process (arrowhead) in the gall bladder. The virtual low monoenergetic image increases the HU of the process; iodine density overlay shows increased levels of iodine around 1 mg/ml; *Z*-effective shows average atomic number close to the liver parenchyma. It could be an adenoma or adenocarcinoma; however, the patient did not wish to have further workup done. **b** Conventional CT show a slight hyperdense process in the gallbladder. Virtual low monoenergetic images make the lesion become hypodense, no iodine density is seen and *Z*-effective images show average atomic numbers closer to fat, giving us the diagnosis of a cholesterol stone

Virtual low monoenergetic series are efficient at detecting gall stones [33], as even cholesterol stones are detected (Fig. 6a). However, it may be difficult to distinguish between some types of gall stones and enhancing polyps on CE-CT. We found significantly more gall bladder polyps by relying on iodine perfusion instead of HU values. To our knowledge, the use of CE-SCT to identify gall bladder polyps has not yet been described in the literature (Fig. 6b).

When CE-SCT are considered, one shall be aware of artifacts that could affect material decomposition, especially in the vicinity of high-density materials in the form of metal implants or high concentration of iodine contrast. These can result in photon starvation leading to a deterioration of image quality on both CE-CT and CE-SCT. An example of such image deterioration can be seen in the electronic supplementary material.

The increase in reading time seems to be very well justified by the improvement in accuracy, confidence level, and a decreased need for follow-up investigations. These improvements will likely lead to a decrease in time to treat, improved patient satisfaction, and general outcome.

Our study has some limitations. First and foremost, we lack histopathological confirmation of some diagnoses; however, for all patients, we have a relatively long median follow-up period of 21.3 months. Inter-observer variability was not performed as the study design was intended to mimic clinical routine as close as possible.

In conclusion, the use of CE-SCT on patients suspected for serious illness that could be cancer increases the confidence of the radiologists in the correct characterization of hypo- and hyperdense lesions in the liver, pancreas, and kidneys; minimizes the need for supplementary examinations at the cost of a minimal increase in reading time; but does not affect the scanner unit workflow dramatically.

## Electronic supplementary material

ESM 1 (DOCX 426 kb)

## Abbreviations

CE-CT: Contrast-enhanced computed tomography
CE-SCT: Contrast-enhanced spectral computed tomography
CT: Computed tomography
DK: Denmark
DLP: Dose length product
GP: General practitioner
HU: Hounsfield units
keV: Kiloelectron volt
kVp: Kilovolt peak
mAs: Milliamperes per second
MRI: Magnetic resonance imaging
NBH: National Board of Health
ROI: Region of interest
VNC: Virtual non-contrast
*Z*_eff_: Effective atomic number
