# Supplementary material for: Impact of spectral body imaging in patients suspected for occult cancer: a prospective study of 503 patients
Source: Eur Radiol. 2020 May 4;30(10):5539–50. doi: 10.1007/s00330-020-06878-7 (PMC7476920; doi:10.1007/s00330-020-06878-7)

**Electronic supplementary material 1** **A)** Box-and-whisker plot describing the reading times (without loading or reporting) for the conventional CT (Mean 300 s, CI95: 291 – 308 s) and the spectral CT (Mean 382 s, CI95: 369 – 395). **B)** The differences in reading times for Spectral CT was lower when the radiologists were certain (Mean 382 s, CI95: 368 – 395 s) in comparison to when there was uncertainty(437 s, CI95: 376 – 499s), showing that some of the outliers in figure 1a is caused by an increase in reading time in difficult cases.


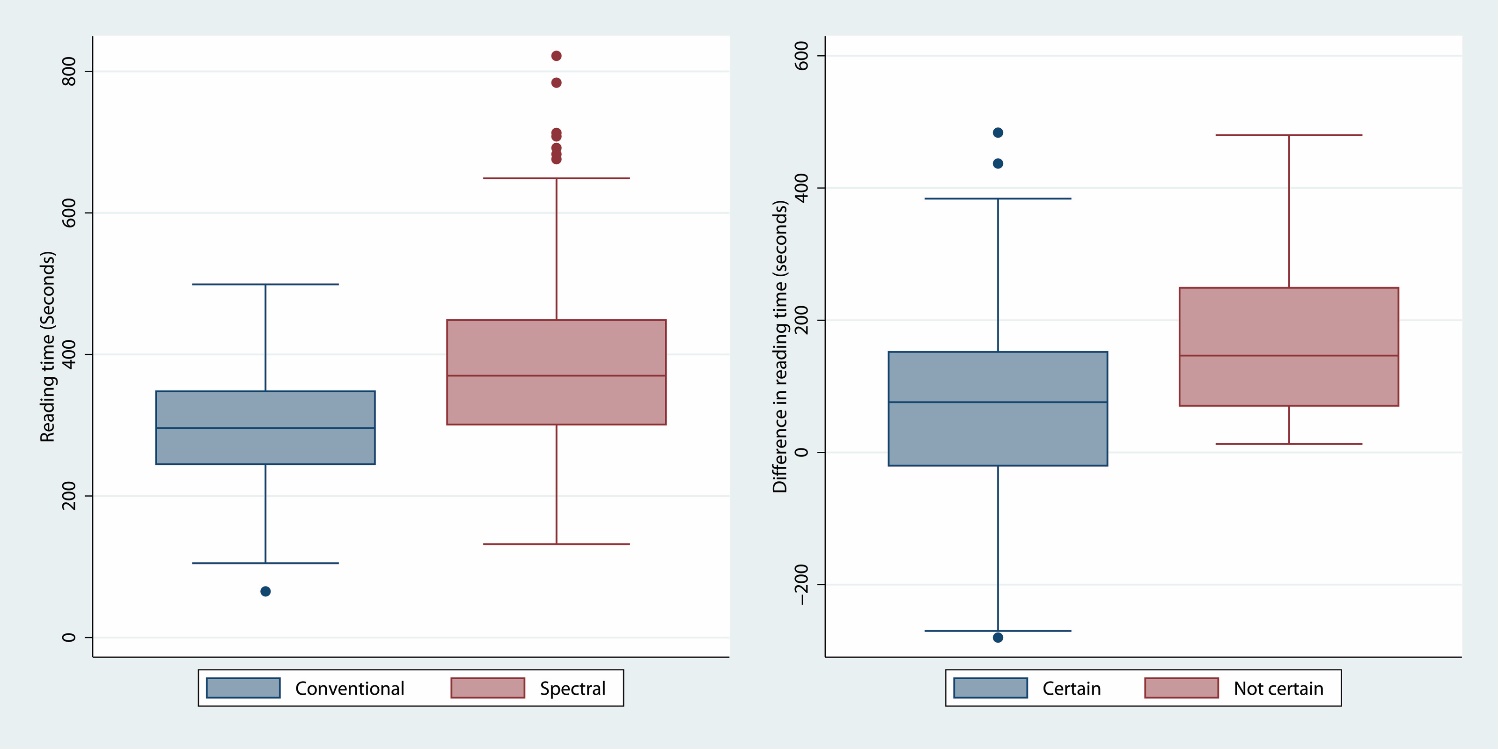


**Electronic supplementary material 2** The conventional image shows a high iodine concentration in v. cava superior and artefacts due to photon starvation, obscuring the tumor in the right hilum. The artefacts are increased in the virtual monoenergetic image and affect calculation of both iodine density and Z-effective values.


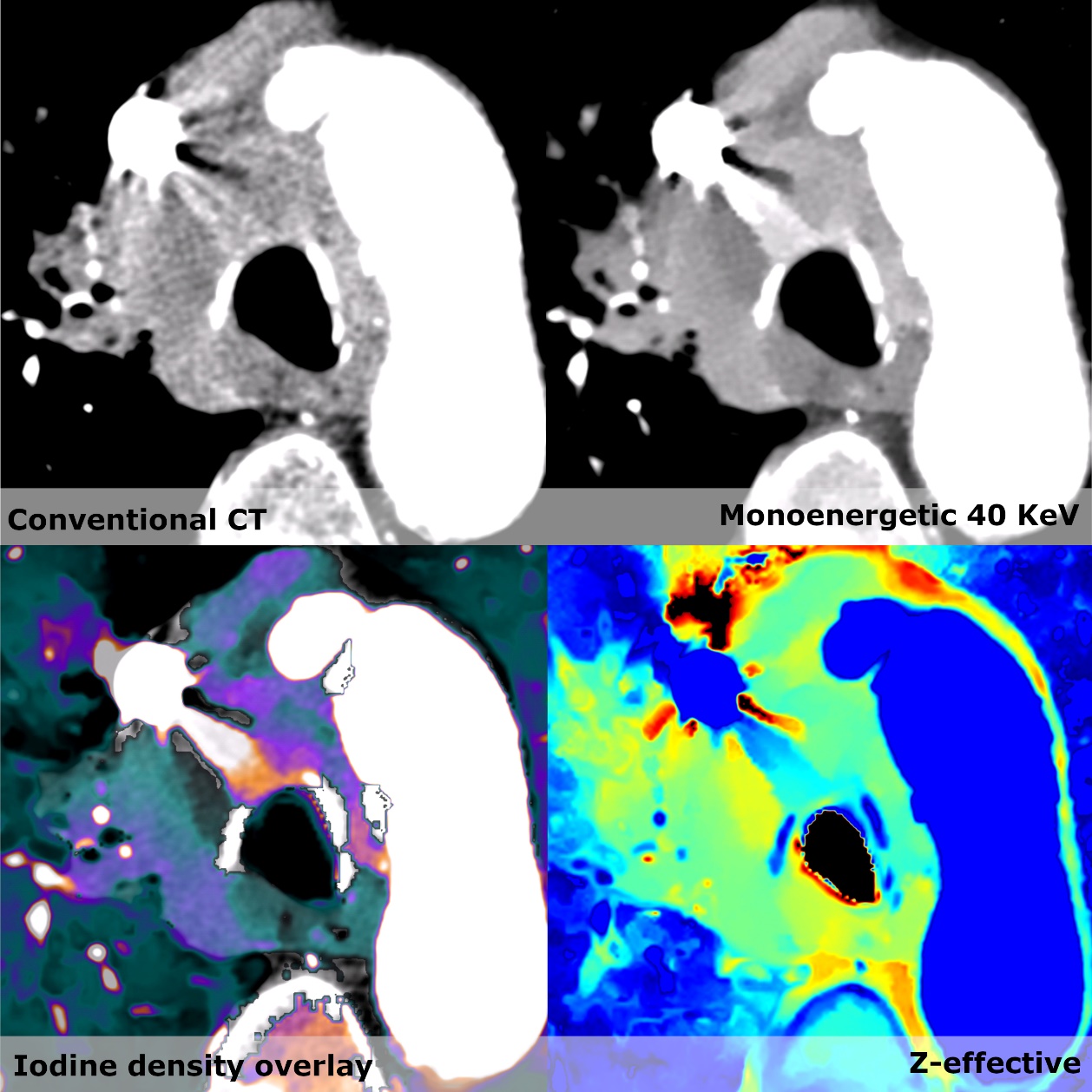

Supplement: Supplementary file 1 — (DOCX 426 kb) [file 330_2020_6878_MOESM1_ESM.docx]
